# Performance, Carcass Yield, Muscle Amino Acid Profile, and Levels of Brain Neurotransmitters in Aged Laying Hens Fed Diets Supplemented with Guanidinoacetic Acid

**DOI:** 10.3390/ani11113091

**Published:** 2021-10-29

**Authors:** Omar A. Ahmed-Farid, Ayman S. Salah, Mohamed Abdo Nassan, Mahmoud S. El-Tarabany

**Affiliations:** 1 Physiology Department, National Organization for Drug Control and Research (NODCAR), Giza P.O. Box 35521, Egypt; ebntaimya@yahoo.com; 2 Department of Animal Nutrition and Clinical Nutrition, Faculty of Veterinary Medicine, New Valley University, El-Kharga P.O. Box 72511, Egypt; asabry3999@yahoo.com; 3 Department of Clinical Laboratory Sciences, Turabah University College, Taif University, P.O. Box 11099, Taif 21944, Saudi Arabia; m.nassan@tu.edu.sa; 4 Department of Animal Wealth Development, Faculty of Veterinary Medicine, Zagazig University, Zagazig P.O. Box 44511, Egypt

**Keywords:** laying hens, guanidinoacetic acid, meat quality, neurotransmitters

## Abstract

**Simple Summary:**

In commercial laying hens, aging is associated with a lower egg production rate and the marketing of spent hen carcasses shows some difficulty, probably due to the expected low meat yield. Using these hens as whole carcasses or to produce meat products for large-scale consumption could provide economic benefits to the poultry markets in developing countries. Thus, the aim of this study was to investigate the effect of dietary supplementation with guanidinoacetic acid (GA) on the carcass yield and muscle amino acid profile of aged laying hens. Dietary GA supplements were shown to improve the carcass yield and the levels of essential amino acids in the breast and thigh muscles of laying hens.

**Abstract:**

Guanidinoacetic acid (GA) is a natural precursor of creatine in the body and is usually used to improve the feed conversion and cellular energy metabolism of broiler chickens. The objective was to elucidate the effect of dietary supplementation of GA on carcass yield, muscle amino acid profile, and concentrations of brain neurotransmitters in laying hens. In total, 128 72-week-old ISA Brown laying hens were assigned to four equal groups (32 birds, eight replicates per group). The control group (T_1_) was fed a basal diet with no supplements, while the other experimental groups were fed a basal diet supplemented with 0.5 (T_2_), 1.0 (T_3_), and 1.5 (T_4_) g GA kg^−1^ diet. The T_3_ and T_4_ groups showed higher hen-day egg production and carcass yield compared to the control group (*p* = 0.016 and 0.039, respectively). The serum creatine level increased linearly with the increased level of dietary GA (*p* = 0.007). Among the essential amino acids of breast muscle, a GA-supplemented diet linearly increased the levels of leucine, isoleucine, phenylalanine, methionine, and threonine in the breast (*p* = 0.003, 0.047, 0.001, 0.001, and 0.015, respectively) and thigh (*p* = 0.026, 0.001, 0.020, 0.009, and 0.028, respectively) muscles. GA supplementation linearly reduced the level of brain serotonin compared to the control group (*p* = 0.010). Furthermore, supplementation of GA in the diet of laying hens linearly increased the level of brain dopamine (*p* = 0.011), but reduced the level of brain Gamma-aminobutyric acid (*p* = 0.027). Meanwhile, the concentration of brain nitric oxide did not differ between the experimental groups (*p* = 0.080). In conclusion, the dietary supplementation of GA may improve the carcass yield and levels of essential amino acids in the breast muscles, as well as the brain neurotransmitters in aged laying hens.

## 1. Introduction

Guanidinoacetic acid (GA) is usually synthesized in the liver and kidneys from glycine and arginine [1]. Moreover, GA is a natural precursor of creatine in the body of vertebrates [2]. Additionally, the creatine contents in the body are irreversibly changed to creatinine, a secretory form of creatine, which is usually eliminated in urine [3]. Hence, the essential requirements of creatine could be provided by endogenous synthesis or from protein sources in the diet. Although a major proportion of creatine requirements can be synthesized by endogenous pathways, 25–33% of the total requirement must be supplemented in a bird’s diet. In this context, there is a promising trend to minimize or eliminate animal protein sources in the diet of poultry. Thus, the addition of GA to the diet of poultry may be crucial for providing the normal requirements of creatine and maintaining the optimum growth performance when birds are fed a protein of vegetable origin [4].

Several trials have evaluated the function of GA as a creatine precursor in broiler chickens [1,2,5], but there is scarce literature on GA supplementation in the diet of laying hens. Indeed, the addition of GA to the diet of broilers improves the feed conversion ratio and cellular energy metabolism [1,2]. At the cellular level, GA has several antioxidative and anti-apoptotic effects [6]. In one of the scarce studies on laying birds, Murakami et al. [7] stated that the addition of GA to the diet of breeders (0.15%) improves the performance of meat-type quails. On the contrary, other authors have suggested that GA supplements is not an efficient strategy to improve the performance of laying hens [8].

It is believed that aging is associated with a reduction in creatine levels in brain tissues [9]. In addition, the dietary supplementation of GA or creatine is vital for maintaining the concentration of creatine in brain tissues, even when the pathway of creatine synthesis in brain is efficient [10]. Based on animal model trials, supplements of creatine or its precursors may be an effective defense mechanism against some neuromuscular [11] and neurodegenerative disorders [12]. Consequently, it is postulated that GA, as a precursor of creatine, may improve the energy metabolism and meat quality of broiler chickens [13].

In recent decades, it was difficult to market the carcasses of spent laying hens or even sell them at a low price [14,15]. Recently, it has been reported that the meat of spent laying hens has a similar nutritional value as seen in commercial chickens [16]. Hence, using these hens as whole carcasses or to produce meat products for large-scale consumption could provide economic benefits to the poultry markets in developing countries [17]. To the best of our knowledge, this is the first trial to explore the effects of GA supplementation on the meat composition and brain neurotransmitters of laying hens. Hence, the present work was designed to evaluate the effects of dose-dependent GA supplementation on the carcass yield, muscle amino acid profile, and brain neurotransmitters of commercial laying birds during the late stage of production.

## 2. Materials and Methods

### 2.1. Birds and Management

In total, 128 aged laying hens of the ISA Brown breed (72 weeks old) were obtained from a commercial flock at a 72.56% hen-day egg production rate. Equally, laying hens were divided into four groups (32 birds, with 8 replicate cages in each group), and housed in wire cages (4 birds/cage). The cage dimensions were 50 cm in length, 46 cm in width, and 42 cm in height. The light regime was 16 h/day and ambient temperature averaged 26 ± 1.5 °C. Throughout the experiment, all hens had free access to water and feed. Throughout a 6-week experimental period, a corn–soybean meal basal diet was fed to meet the nutritional requirement of laying hens [18] (Table 1). The control group were fed the basal diets with no supplements (T_1_). The other experimental groups were fed basal diets supplemented with 0.5 (T_2_), 1.0 (T_3_), and 1.5 (T_4_) g GA kg^−1^ diet. The GA supplements were purchased from Evonic Inc. (CreAmino^®^; 99% guanidinoacetic acid).

### 2.2. Laying Performance 

The daily hen-day egg production rate (HDEP) was recorded for all groups. On a replicate basis, feed intake was reported. Moreover, the feed conversion ratio (FCR) was calculated as feed consumption (g)/eggs produced (g).

### 2.3. Blood Sampling and Serum Biochemical Parameters

At 78 weeks of age, two birds were selected from each cage (16 birds/group) to collect 3 mL blood samples via the brachial vein route. In order to separate sera, blood samples were centrifuged as quickly as possible (1008× *g*) and then stored at −20 °C. The concentrations of serum creatine and alanine aminotransferase (ALT) were with commercial Roche diagnostics kits (GmbH, Mannheim, Germany).

### 2.4. Carcass Yield and Muscle Amino Acid Profile

At the end of this trial, 8 birds from each group were randomly chosen and slaughtered according to the Islamic protocol (HALAL Slaughter) of Malaysian institutes [19]. The main jugulars of the birds were severed with sharp knives without using any anesthetic to achieve effective bleeding. After evisceration, the carcasses were chilled (2 °C for 30 min). The carcass yield (dressing percentage) was estimated as an actual carcass weight relative to the live body weight. The breast and thigh muscles were dissected from each carcass, with careful removal of connective tissues. The amino acid profiles in the breast muscles were determined [20]. The visible external fat was removed and the meat sample (1 g) was mixed with 10 mL of 2% trichloroacetic acid solution, homogenized at 16,128× *g* for 1 min, and then centrifuged at 2800× *g* for 10 min. The derivatizing agent was added, which consisted of a methanol/TEA/deionized water/phenylisothiocyanate mixture (7:1:1:1 mL). After derivatization and drying, the samples were mixed with a diluent composed of 0.71 g of. disodium-hydrogen phosphate (pH 7.4) plus 5% acetonitrile. The prepared samples and AA standards were injected into a Nova-PakTM C18 column (4 µm, 3.9 mm × 4.6 mm) for separation and quantification of free AA by HPLC (Agilent HP 1200 series apparatus, Santa Clara, CA, USA). Amino acids were separated according to a gradient mobile phase composed of buffer A (50 mol/L ammonium acetate buffer, pH 6.5) and buffer B (100 mol/L ammonium acetate acetonitrile, 50:50 mL, pH 6.5).

### 2.5. Nitric Oxide and Monoamine Concentrations in Brain Tissues

After stunning the chosen birds, the brain samples (striatum, frontal cortex, and hypothalamus) were homogenized in 75% aqueous HPLC-grade methanol (10% *w*/*v*) [21]. The derivatization process started by re-drying the samples using a solution consisting of a 2:2:1 mixture of methanol/1 M sodium acetate trihydrate/triethylamine (TEA). The homogenate of each sample was centrifuged at 1792× *g* for 10 min and the supernatant part was divided into two equal volumes; the first part was dried at room temperature for amino acid determination, whereas the second portion was used for monoamine evaluation. The levels of brain monoamines (microgram per gram of brain tissue) were measured by HPLC [22]. The brain nitric oxide concentration was determined according to the modified method of Papadoyannis et al. [23].

### 2.6. Statistical Analyses

The data were analyzed by ANOVA procedures of the IBM SPSS software program (version 16.0; IBM Corp., Armonk, NY, USA). For all variables, pen was considered as the replicate (experimental unit). The orthogonal polynomials for diet responses were determined by linear and quadratic effects. All results are expressed as means and the residual standard deviation (RSD). The statistical model included the following effects:

Y_ij_ = μ + T_i_ + e_ij_

where,

Y_ij_ = the dependent variable;
μ = the population mean;
T_i_ = the fixed effect of GA dietary supplements (i = T_1_, T_2_, T_3_, and T_4_);
e_ij_ = random error, assumed to be normally and independently distributed.

## 3. Results

**Figure 1:**
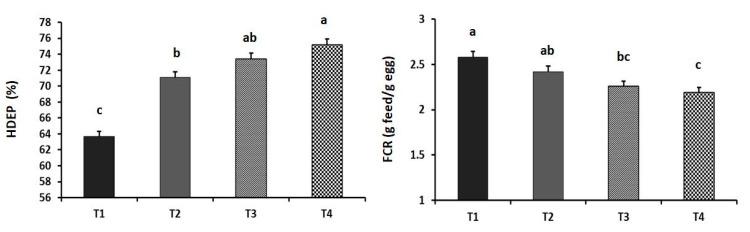
Effect of the dietary supplementation of guanidinoacetic acid (GA) on hen-day egg production (HDEP) (*p* = 0.016 and 0.873 as linear and quadratic, respectively) and feed conversion ratio (*p* = 0.018 and 0.452 as linear and quadratic, respectively) of commercial laying hens at the late stage of production. T_1_ = control group; T_2_ = group supplemented with 0.5 g GA kg^−1^ diet; T_3_ = group supplemented with 1 g GA kg^−1^ diet; T_4_ = group supplemented with 1.5 g GA kg^−1^ diet. Means with different letters (a, b, and c) significantly differ at *p* ˂ 0.05.

**Figure 2:**
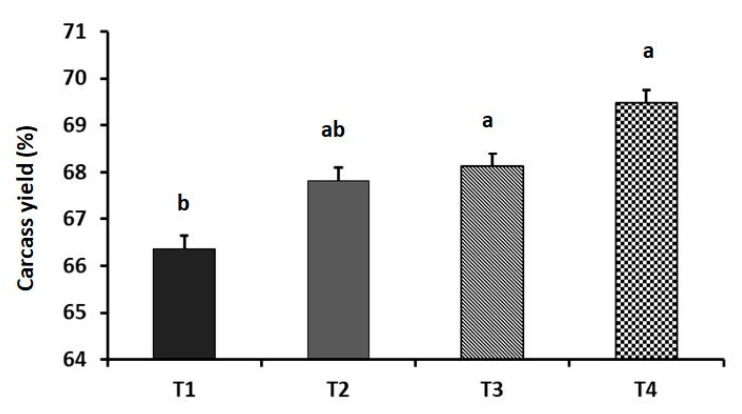
Effect of dietary supplementation of guanidinoacetic acid (GA) on the carcass yield of aged laying hens (*p* = 0.039 and 0.590 as linear and quadratic, respectively). T_1_ = control group; T_2_ = group supplemented with 0.5 g GA kg^−1^ diet; T_3_ = group supplemented with 1 g GA kg^−1^ diet; T_4_ = group supplemented with 1.5 g GA kg^−1^ diet. Means with different letters (a, b) significantly differ at *p* ˂ 0.05. *n* = 8 birds/group.

**Figure 3:**
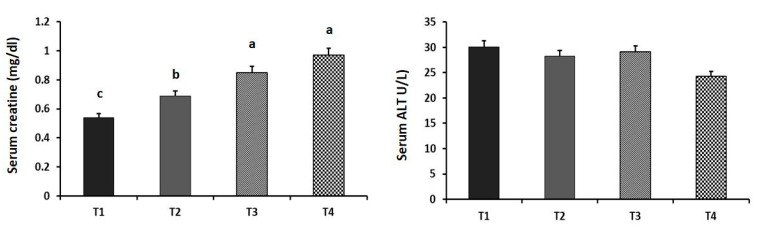
Effect of dietary supplementation of guanidinoacetic acid (GA) on the serum creatine (*p* = 0.007, 0.063, and 0.018 as linear, quadratic, and cubic, respectively) and alanine aminotransferase (ALT) levels of aged laying hens (*p* = 0.521, 0.090, and 0.658 as linear, quadratic, and cubic, respectively). T_1_ = control group; T_2_ = group supplemented with 0.5 g GA kg^−1^ diet; T_3_ = group supplemented with 1 g GA kg^−1^ diet; T_4_ = group supplemented with 1.5 g GA kg^−1^ diet. Means with different letters (a, b, and c) significantly differ at *p* ˂ 0.05. *n* = 16 birds/group.

The effects of dietary GA supplementation on the performance and blood chemistry of laying hens are illustrated in Figure 1, Figure 2 and Figure 3. The HDEP increased linearly with the dietary levels of GA (*p* = 0.016). The T_4_ group showed the highest HDEP (75.17%). Furthermore, laying hens in the T_3_ and T_4_ groups had better FCR than did the GA_0_ group (*p* = 0.018). Birds in the T_3_ and T_4_ groups showed a significantly higher carcass yield (69.48% and 68.13%, respectively) compared to the control group (*p* = 0.039). Although the serum ALT level was not affected (*p* = 0.521) by the dietary supplements, the serum creatine level was increased linearly with the increased inclusion level of GA in the diet (*p* = 0.007).

As described in Table 2 and Table 3, supplementation of GA linearly increased the levels of leucine, isoleucine, phenylalanine, methionine, and threonine in the breast (*p* = 0.003, 0.047, 0.001, 0.001, and 0.015, respectively) and thigh (*p* = 0.026, 0.001, 0.020, 0.009, and 0.028, respectively) muscles. Furthermore, dietary GA supplementation linearly improved the contents of non-essential AAs such as arginine, glutamine, proline, histidine, and taurine in the breast (*p* = 0.001, 0.016, 0.003, 0.012, and 0.016, respectively) and thigh (*p* = 0.022, 0.001, 0.004, 0.019, and 0.025, respectively) muscles. Meanwhile, dietary GA supplementation linearly decreased the levels of lysine in the breast (*p* = 0.013) and thigh (*p* = 0.003) muscles.

As illustrated in Table 4, GA supplementation linearly reduced the level of brain serotonin compared to the control group (*p* = 0.010). Furthermore, supplementation of GA in the diet of laying hens linearly increased the brain dopamine level (*p* = 0.011), but reduced the level of brain GABA (*p* = 0.027). Meanwhile, the current trial did not reveal significant differences in the levels of brain nitric oxide, histidine, or glutamate.

## 4. Discussion

Although some authors have demonstrated that dietary GA supplementation results in a limited improvement in the egg production rate of meat-type quail breeders and laying hens [7,8,24], the current study reported that an increased dietary level of GA is associated with a significantly linear increase in the HDEP in commercial laying hens. In this context, GA could compensate for the arginine in the diet of poultry [2]. In vertebrates, the crucial role of arginine in protein and nitric oxide synthesis has been confirmed [25]. Indeed, nitric oxide stimulates the pituitary gland to release GnRH, with subsequent control of the activity of FSH and LH hormones [26]. Concomitantly, dietary GA supplementation increases the levels of LH and FSH in commercial laying hens [8]. Basiouni et al. [27] also reported that the addition of 1.5% digestible arginine to the diet of hens increases the egg production rate by 15.8%. The GA-supplemented groups herein showed better FCR than did the control group. In broiler chickens, dietary GA supplementation improves the FCR [28]. Meanwhile, other authors have found that GA-supplemented diets do not improve the FCR in laying hens [8] or broilers [29].

Herein, the carcass yields of aged laying hens linearly increased with increased dietary GA supplementation. Consistent with these findings, several authors have stated that the supplementation of GA improves the breast meat yield in birds fed a vegetable-based diet [2;4]. Additionally, Esser et al. [30] reported that heat-stressed birds fed GA-supplemented diets have a greater breast yield than do the birds in the control group. These positive effects of GA supplementation may be attributed to creatine, the metabolic end product of GA, which plays a crucial role in the regulation of the energy-buffering system in muscles, as well as optimization of the protein metabolism [31]. In this sense, Michiels et al. [2] reported that the supplementation of GA markedly increases the concentration of creatine in the breast muscles of broiler chickens. Another hypothesis suggests that the action of GA is mainly associated with the metabolic pathway of amino acids. Indeed, GA supplementation may spare arginine, which is one of the potentially deficient AAs in vegetable-based diets [24]. On the contrary, some recent trials showed that the addition of GA to the diet of broilers does not improve carcass yields or other quality traits [32,33].

The current study demonstrated positive effects of dietary GA supplementation on the levels of EAAs (leucine, phenylalanine, threonine, and methionine) and non-essential AAs (arginine, glutamine, proline, histidine, and taurine) in the breast and thigh muscles of aged laying hens. It has been reported that broiler chickens have superior meat quality parameters compared to spent laying hens [34]. However, it is important to use the meat of laying hens at the end of the laying cycle as a source of animal protein in developing countries [35]. In this context, EAAs are considered key parameters in food quality assessment for human consumption [36]. The concentrations of EAAs in the breast muscles of the control group were clearly lower than those reported in broiler breast muscles [37]. Interestingly, the concentrations of most EAAs (leucine, isoleucine, phenylalanine, threonine, and methionine) in the breast muscles of the T_3_ and T_4_ groups were nearly equal to or greater than those in the breast muscles of Ross broiler chickens [35,38]. This could be attributed to the auxiliary role of GA in cellular bioenergetics and the adjustment of oxidant–antioxidant status, probably by stimulating in vivo creatine synthesis [33]. It has also been suggested that the higher methionine contents in the breast muscles may be attributed to the ability of GA supplementation to conserve methionine amino acids [39]. Additionally, the contents of arginine and glutamine in the breast muscles of the T_4_ group were relatively greater than those in the breast muscles of broiler chickens. Interestingly, the current study demonstrated that dietary GA supplementation linearly reduces the contents of lysine in the breast and thigh muscles of laying hens. Although there is no information available to explain these results, a previous trial recorded that dietary humic acids decrease the level of lysine in the breast muscles of broiler chickens [40].

Although the serum ALT level was not affected by dietary supplementation of GA, the serum creatine level linearly increased in the GA-supplemented groups. Consistent with these findings, Ostojica et al. [39] reported that the oral supplementation of GA increases the creatine concentration in the serum of young healthy volunteers. In a two-week trial, rats fed a GA-supplemented diet showed a 6-fold higher serum creatine level [41]. In this context, the European Food Safety Authority [42] summarized the research on the efficacy of GA as a feed additive in the poultry industry. The data showed an increased serum creatine content after supplementing the diet of broilers with GA for 42 days. The increased creatine concentration in the blood with an increased dietary GA level may reflect several metabolic changes in the liver and muscles and suggests increased transport of the metabolites to excretion organs [29]. Contrary to our findings, others have suggested that different levels of dietary GA do not influence the serum creatine concentrations in broilers [43] or piglets [44]. 

Next to its role in cellular bioenergetics, it has been postulated that GA might have further physiological roles such as activation of hormonal release and neuromodulation [45]. Indeed, serotonin is an important neurotransmitter involved in the normal functional activity of the brain and plays a significant role in controlling the contractility of gastrointestinal smooth muscles [46]. The current study revealed that the control group showed a significantly higher brain serotonin concentration than did the GA-supplemented groups. Considering that serotonin may exert an inhibitory action on feeding in chickens [47], GA supplementation may improve the feeding patterns and consequent carcass yield of laying hens. While some researchers have reported that an injection of a serotonin agonist reduces the food intake in the fasted–refed birds [48], others have reported an inhibitory effect on feeding patterns in fed and fasted adult quails [49].

Chemically, dopamine is a catecholamine neurotransmitter, which plays a crucial role in the normal activity of the central nervous system [50]. Herein, dietary GA supplementation linearly increased the concentration of dopamine in the brain tissues of laying hens. In this context, Sartsoongnoen et al. [51] suggested that dopaminergic neurons are involved in the reproductive regulatory system in Thai laying hens. In addition to any localized regulation within the gastrointestinal tract, it has been suggested that DA may play a role in “gut–brain” axis regulation [52]. In a recent study, Li et al. [53] suggested that dietary supplementation with l-theanine improves the dopamine levels in the brain of adult rats. However, others have reported that laying hens selected for improved productivity and survivability have lower circulating concentrations of dopamine than those selected for low productivity and short survivability [31]. 

Gamma-aminobutyric acid (GABA) is an essential inhibitory neurotransmitter in the CNS [54]. Furthermore, it has been suggested that GA probably acts as a modulator of GABA metabolism in brain and peripheral tissues [55]. The current study revealed a significant reduction in the concentration of GABA in the brain tissues of GA-supplemented groups. Consistent with our findings, Ostojic and Stojanovic [56] reported that GA supplementation down-regulates GABA synthesis in peripheral tissues. On the contrary, others have stated that GA could act as an activator of GABA receptors in the brain and peripheral tissues, with possible effects on the muscular tone or brain development [57]. L- histidine is one of the essential precursors to synthesize carnosine, a dipeptide molecule that has antioxidative activity in brain and skeletal muscles. Although the current research demonstrated a high concentration of histidine in the breast muscles of GA-supplemented hens, dietary GA supplementation did not affect the concentration of histidine in brain tissues.

## 5. Conclusions

From the aforementioned findings, it could be concluded that dietary supplementation of GA may improve the carcass yield and levels of essential AAs in the breast muscles of laying hens. Moreover, dietary GA supplementation at doses of 1 or 1.5 g/kg may improve the activity of some monoamine neurotransmitters (serotonin, dopamine, and GABA) in the brain tissues of spent laying hens.

## Figures and Tables

**Table 1:** Ingredients, composition, and calculated chemical analysis of the basal diets.

	g/kg DM
Ingredients	
Yellow maize	602.0
Soybean meal (44%)	260.0
Limestone	87.0
Dicalcium phosphate	17.0
Sodium bicarbonate	2.3
DL-methionine	1.3
Vitamin and trace mineral mix	3.0
NaCl	2.4
Maize oil	25.0
Calculated analysis	
ME (KJ/kg)	12,029
Crude protein	166.0
Calcium	37.7
Available phosphorus	4.5
Lysine	8.5
Leucine	12.8
Isoleucine	6.7
Arginine	9.4
Methionine	3.9
Methionine + cysteine	6.3
Tryptophan	2.2
Threonine	6.1
Phenylalanine	7.8
Histidine	4.3
Valine	7.7

DM, dry matter; ME, metabolizable energy.

**Table 2:** Effect of dietary supplementation with GA on the amino acid profile (g 100 g^−1^) of the breast muscles in laying hens.

Item	Experimental Groups					Contrast		
	^1^ T_1_	^2^ T_2_	^3^ T_3_	^4^ T_4_	^5^ RSD	Linear	Quadratic	Cubic
Lysine	84.4 ^a^	77.5 ^ab^	73.9 ^b^	72.3 ^b^	6.04	0.013	0.245	0.308
Leucine	45.8 ^c^	59.3 ^b^	60.3 ^b^	71.1 ^a^	4.49	0.003	0.227	0.026
Isoleucine	30.4 ^b^	38.7 ^a^	38.7 ^a^	39.1 ^a^	2.44	0.047	0.087	0.178
Phenylalanine	15.7 ^c^	19.2 ^b^	21.1 ^a^	25.7 ^a^	1.68	0.001	0.045	0.018
Valine	33.3	41.9	42.7	43.7	3.71	0.073	0.257	0.416
Threonine	26.4 ^b^	33.9 ^ab^	30.2 ^ab^	37.7 ^a^	3.60	0.015	0.150	0.102
Methionine	13.9 ^c^	17.8 ^b^	17.1 ^bc^	21.4 ^a^	1.52	0.001	0.824	0.055
Serine	21.8	28.5	27.8	28.8	2.86	0.098	0.390	0.374
Aspartic acid	62.6 ^b^	66.2 ^ab^	71.4 ^ab^	85.6 ^a^	8.44	0.010	0.707	0.156
Glutamine	67.9 ^c^	85.5 ^b^	91.2 ^b^	122.9 ^a^	9.60	0.016	0.180	0.220
Proline	13.3 ^b^	16.5 ^ab^	17.1 ^ab^	20.3 ^a^	1.47	0.003	0.832	0.034
Alanine	35.5 ^b^	44.6 ^ab^	38.7 ^ab^	47.9 ^a^	3.34	0.019	0.229	0.076
Arginine	54.3 ^b^	51.5 ^b^	65.6 ^a^	65.2 ^a^	4.48	0.001	0.225	0.013
Histidine	32.5 ^b^	36.9 ^ab^	40.6 ^a^	42.1 ^a^	3.16	0.012	0.296	0.030
Glycine	28.3	35.5	35.9	36.7	2.08	0.087	0.303	0.219
Tyrosine	25.3 ^b^	35.1 ^a^	31.9 ^ab^	37.8 ^a^	3.27	0.004	0.118	0.087
Taurine	69.9 ^c^	85.8 ^bc^	93.7 ^ab^	109.7 ^a^	6.46	0.016	0.319	0.022

^1^ Control group; ^2^ group supplemented with 0.5 g GA kg^−1^ diet; ^3^ group supplemented with 1 g GA kg^−1^ diet; ^4^ group supplemented with 1.5 g GA kg^−1^ diet; ^5^ residual standard deviation. Different superscript letters within each row are significantly different at *p* ˂ 0.05. *n* = 8 birds/group.

**Table 3:** Effect of dietary supplementation with GA on the amino acid profile (g 100 g^−1^) of the thigh muscles in laying hens.

Item	Experimental Groups					Contrast		
	^1^ T_1_	^2^ T_2_	^3^ T_3_	^4^ T_4_	^5^ RSD	Linear	Quadratic	Cubic
Lysine	52.7 ^a^	53.7 ^a^	44.8 ^b^	36.7 ^c^	2.37	0.003	0.036	0.001
Leucine	35.3 ^b^	43.5 ^ab^	44.5 ^ab^	53.2 ^a^	2.97	0.026	0.417	0.193
Isoleucine	28.3 ^b^	35.7 ^a^	33.3 ^ab^	37.1 ^a^	2.06	0.001	0.139	0.014
Phenylalanine	16.5 ^b^	22.1 ^ab^	20.8 ^b^	27.2 ^a^	1.41	0.020	0.076	0.029
Valine	23.8 ^b^	29.9 ^ab^	33.2 ^ab^	37.8 ^a^	3.52	0.035	0.676	0.491
Threonine	20.5 ^b^	26.9 ^a^	23.3 ^ab^	27.9 ^a^	2.18	0.028	0.369	0.123
Methionine	9.6 ^c^	11.6 ^bc^	12.2 ^ab^	14.5 ^a^	1.79	0.009	0.561	0.292
Serine	17.1 ^c^	21.9 ^b^	24.2 ^ab^	27.1 ^a^	2.13	0.012	0.255	0.394
Aspartic acid	44.1 ^b^	56.4 ^a^	59.6 ^a^	56.1 ^a^	4.75	0.010	0.031	0.753
Glutamine	68.8 ^c^	86.2 ^b^	105.9 ^a^	111.5 ^a^	6.84	0.001	0.065	0.233
Proline	13.2 ^c^	16.7 ^bc^	18.4 ^b^	25.6 ^a^	1.67	0.004	0.039	0.062
Alanine	28.5 ^c^	35.8 ^bc^	37.5 ^ab^	45.2 ^a^	2.36	0.008	0.630	0.142
Arginine	35.6 ^c^	34.2 ^c^	40.9 ^b^	46.8 ^a^	2.32	0.022	0.025	0.032
Histidine	18.1 ^b^	21.9 ^b^	19.7 ^b^	29.9 ^a^	1.64	0.019	0.020	0.004
Glycine	22.4 ^b^	30.4 ^a^	30.3 ^a^	36.9 ^a^	2.37	0.036	0.586	0.020
Tyrosine	13.8 ^b^	17.9 ^b^	17.4 ^b^	23.2 ^a^	1.70	0.024	0.414	0.031
Taurine	56.4 ^b^	74.3 ^ab^	67.6 ^b^	92.2 ^a^	7.87	0.025	0.454	0.009

^1^ Control group; ^2^ group supplemented with 0.5 g GA kg^−1^ diet; ^3^ group supplemented with 1 g GA kg^−1^ diet; ^4^ group supplemented with 1.5 g GA kg^−1^ diet; ^5^ residual standard deviation. Different superscript letters within each row are significantly different at *p* ˂ 0.05. *n* = 8 birds/group.

**Table 4:** Effect of dietary supplementation with GA on the levels of nitric oxide and neurotransmitters in the brain tissues of laying hens.

Parameter	Experimental Groups					Contrast		
	^1^ T_1_	^2^ T_2_	^3^ T_3_	^4^ T_4_	^5^ RSD	Linear	Quadratic	Cubic
Nitric oxide (μmol/g)	25.8	24.9	26.6	31.4	2.68	0.080	0.132	0.216
Dopamine (μg/g)	1.02 ^b^	1.03 ^b^	1.27 ^a^	1.48 ^a^	0.101	0.011	0.131	0.006
Serotonin (μg/g)	0.76 ^a^	0.57 ^b^	0.61 ^b^	0.49 ^b^	0.065	0.010	0.019	0.042
^6^ GABA (μg/g)	8.12 ^a^	7.33 ^ab^	6.32 ^bc^	5.41 ^c^	0.689	0.027	0.017	0.001
Glutamate (μg/g)	4.16	3.92	3.39	3.44	0.592	0.592	0.321	0.460
Aspartic acid (μg/g)	4.47 ^a^	3.63 ^ab^	3.35 ^b^	3.62 ^ab^	0.494	0.019	0.011	0.086
Histidine (μg/g)	0.46	0.43	0.52	0.71	0.041	0.102	0.243	0.016

^1^ Control group; ^2^ group supplemented with 0.5 g GA kg^−1^ diet; ^3^ group supplemented with 1 g GA kg^−1^ diet; ^4^ group supplemented with 1.5 g GA kg^−1^ diet; ^5^ residual standard deviation, ^6^ Gamma-aminobutyric acid. Different superscript letters within each row are significantly different at *p* ˂ 0.05. *n* = 8 birds/group.

## Data Availability

Data sharing is not applicable. All data analyzed during this study are included in this published paper.
